# The Impact of Normal Saline or Balanced Crystalloid on Plasma Chloride Concentration and Acute Kidney Injury in Patients With Predicted Severe Acute Pancreatitis: Protocol of a Phase II, Multicenter, Stepped-Wedge, Cluster-Randomized, Controlled Trial

**DOI:** 10.3389/fmed.2021.731955

**Published:** 2021-10-04

**Authors:** Bo Ye, Mingfeng Huang, Tao Chen, Gordon Doig, Bin Wu, Mingzhi Chen, Shumin Tu, Xiaomei Chen, Mei Yang, Guoxiu Zhang, Qiang Li, Xinting Pan, Lijuan Zhao, Honghai Xia, Yan Chen, Lu Ke, Zhihui Tong, Rinaldo Bellomo, John Windsor, Weiqin Li

**Affiliations:** ^1^Department of Critical Care Medicine, Jinling Hospital, Nanjing University School of Medicine, Nanjing, China; ^2^Global Health Trials Unit, Department of Clinical Sciences, Liverpool School of Tropical Medicine, Liverpool, United Kingdom; ^3^Northern Clinical School, Royal, North Shore Hospital, University of Sydney, Sydney, NSW, Australia; ^4^Department of General Intensive Care Unit, The Third Hospital of Xiamen City, Xiamen, China; ^5^Department of Critical Care Medicine, Jinjiang Hospital of Traditional Chinese Medicine, Jinjiang, China; ^6^Department of Emergency, The First Hospital of Shangqiu City, Shangqiu, China; ^7^Department of Critical Care Medicine, Qilu Hospital of Shandong University, Jinan, China; ^8^Department of Intensive Care Unit, The Qujing NO.1 People's Hospital, Qujing, China; ^9^Department of Emergency Intensive Care Unit, The First Affiliated Hospital of Henan University of Science and Technology, Luoyang, China; ^10^Pancreas Center, The First Affiliated Hospital of Nanjing Medical University, Nanjing, China; ^11^Department of Emergency Intensive Care Unit, The Affiliated Hospital of Qingdao University, Qingdao, China; ^12^Department of Emergency Intensive Care Unit, First People's Hospital of Yunnan Province, Kunming, China; ^13^Department of Emergency, The First Affiliated Hospital of the University of Science and Technology of China, Hefei, China; ^14^National Institute of Healthcare Data Science at Nanjing University, Nanjing, China; ^15^Department of Critical Care, The University of Melbourne, Melbourne, VIC, Australia; ^16^Australian and New Zealand Research Center, School of Public Health and Preventive Medicine, Monash University, Melbourne, VIC, Australia; ^17^Department of Intensive Care, Austin Hospital, Melbourne, VIC, Australia; ^18^Department of Intensive Care, Royal Melbourne Hospital, Melbourne, VIC, Australia; ^19^Surgical And Translational Research Center, Faculty of Medical and Health Sciences, University of Auckland, Auckland, New Zealand

**Keywords:** acute pancreatitis, saline, crystalloid, acute kidney injury, intravenous fluid

## Abstract

**Introduction/aim:** The supraphysiologic chloride concentration of normal saline may contribute to acute kidney injury (AKI). Balanced crystalloids can decrease chloride concentration and AKI in critically ill patients. We aim to test the hypothesis that, in patients with predicted severe acute pancreatitis (pSAP), compared with saline, fluid therapy with balanced crystalloids will decrease plasma chloride concentration.

**Methods/Design:** This is a multicenter, stepped-wedge, cluster-randomized, controlled trial. All eligible patients presenting to the 11 participating sites across China during the study period will be recruited. All sites will use saline for the first month and sequentially change to balanced crystalloids at the pre-determined and randomly allocated time point. The primary endpoint is the plasma chloride concentration on day 3 of enrollment. Secondary endpoints will include major adverse kidney events on hospital discharge or day 30 (MAKE 30) and free and alive days to day 30 for intensive care admission, invasive ventilation, vasopressors, and renal replacement therapy. Additional endpoints include daily serum chloride and sequential organ failure assessment (SOFA) score over the first seven days of enrollment.

**Discussion:** This study will provide data to define the impact of normal saline vs. balanced crystalloids on plasma chloride concentration and clinical outcomes in pSAP patients. It will also provide the necessary data to power future large-scale randomized trials relating to fluid therapy.

**Ethics and Dissemination:** This study was approved by the ethics committee of Jinling Hospital, Nanjing University (2020NZKY-015-01) and all the participating sites. The results of this trial will be disseminated in peer-reviewed journals and at scientific conferences.

**Trial registration:** The trial has been registered at the Chinese Clinical Trials Registry (ChiCTR2100044432).

## Introduction

The most important determinant of mortality in patients with acute pancreatitis (AP) is organ failure (1). Renal failure or acute kidney injury (AKI) occurs in 25–59% of patients with severe AP (2, 3). It may be due to several factors, including hypovolemia, hypoxemia, pancreatic proteolytic enzymes, impairment of renal microcirculation, decreased renal perfusion pressure, tissue edema, and/or intrabdominal hypertension (4–6). Maintaining normovolaemia by timely and adequate fluid therapy to preserve organ perfusion is considered the cornerstone of the management of AP in the acute phase (7–9).

Normal saline (NS or 0.9% sodium chloride) is the most frequently used isotonic crystalloid fluid for resuscitation in the acute setting around the world (10). Because the chloride concentration of NS (154 mmol per liter) is significantly higher than plasma chloride concentration (94–111 mmol per liter), there is a risk of hyperchloremic metabolic acidosis. The risk of this increases with systemic inflammation and impaired renal perfusion through multiple mechanisms (11). However, the clinical impact of the increased chloride load with NS resuscitation is not fully understood. There is some evidence that it increases the risk of AKI and the need for renal replacement therapy (RRT) in critically ill patients (12, 13), but there is no reliable data in AP patients.

Balanced crystalloid fluids are an alternative to NS, and some current guidelines recommend Lactated Ringers (LR) for early resuscitation, but there is a lack of high-quality evidence (7, 8, 14). Previous studies showed that LR resuscitation can decrease inflammatory markers but did not improve clinical outcomes in patients with predominantly mild AP (15–17). These studies did not report changes in plasma chloride or the incidence of AKI. Our previous study showed that aggressive resuscitation and increased chloride load during the first 24 hours were risk factors for new-onset AKI in patients with moderately severe and severe AP (18). This study aims to compare the impact of NS and a balanced crystalloid fluid on the plasma chloride concentration and the incidence of AKI in patients with predicted severe AP (pSAP).

## Methods and Analysis

### Aim and Objectives

The primary objective of the CLEVER-AP trial is to determine whether, compared to NS, balanced crystalloids significantly reduce plasma chloride concentration in patients with pSAP. The secondary objective is to determine whether a lower plasma chloride concentration is associated with reduced renal dysfunction and improved clinical outcomes.

### Study Design

The present study is an investigator-initiated, stepped-wedge, cluster-randomized trial, in which each participating hospital will begin in the control phase (patients receiving NS) and transition to the intervention phase (patients receiving balanced crystalloids) at randomly assigned time points (wedge). This trial was registered at the Chinese Clinical Trials Registry (ChiCTR2100044432).

### Trial Committees

A Trial Management Committee (TMC) was formed comprising the primary investigators, supported by all the other co-investigators (clinical and non-clinical) from the participating sites and members of the Chinese Acute Pancreatitis Clinical Trials Group (CAPCTG) coordinating center. The TMC is responsible for the day-to-day running and management of the trial. An expert clinical panel consisting of experts from the CAPCTG was organized to provide governance and audit the study (some are not from the participating sites). This panel will also assist with crucial clinical decision-making, such as the introduction of RRT.

A writing and publication committee is responsible for drafting the manuscript and submission of the manuscript to proper academic journals. It will also decide on the authorship of this study.

### Study Population

All adult patients with AP admitted to the participating sites will be assessed for eligibility after admission. This trial will include all patients from the 11 hospitals who met the eligibility criteria during the enrollment period.

### Eligibility Criteria

The inclusion and exclusion criteria are as follows:

#### Inclusion Criteria

1. Diagnosis of acute pancreatitis (19): symptoms and signs of acute pancreatitis based on abdominal pain, serum amylase at least three times the upper limit of the normal range, and/or characteristic findings on computed tomography;
2. Predicted severe acute pancreatitis, based on APACHE-II score≥8 and CRP>150 mg/L.
3. Recruitment within 72 h from the onset of the symptoms;
4. Age between 18 to 70 yrs old;

#### Exclusion Criteria

1. Patients with chronic renal disease [All patients with an eGFR<60 ml/min/1.73 m^2^ for three months are defined as having chronic kidney disease (20)].
2. Patients who need emergent RRT at the time of admission. The indication for RRT will be according to the criteria described by Bellomo et al. (21).
3. Patients who are pregnant or lactating at the time of enrollment.
4. Patients who undergo RRT before admission.
5. Patients receiving percutaneous or transmural drainage for pancreatic necrotic collections or surgery before admission or requiring emergency surgery due to abdominal compartment syndrome, bowel ischemia, etc., at admission.
6. Patients present with fulminant multiple organ failure with predicted death within 24–48 hrs (e.g., severe respiratory failure, severe systemic circulatory failure, coma, or other dangerous symptoms that are difficult to reverse).
7. Patients with a known history of severe cardiovascular, respiratory, renal, hepatic, hematologic, or immunologic disease defined as (1) greater than New York Heart Association class II heart failure, (2) active myocardial ischemia, or (3) cardiovascular intervention within previous 60 days, (4) history of cirrhosis or (5) severe chronic obstructive pulmonary disease requiring home oxygen.

### Randomization Methods

**Figure 1:**
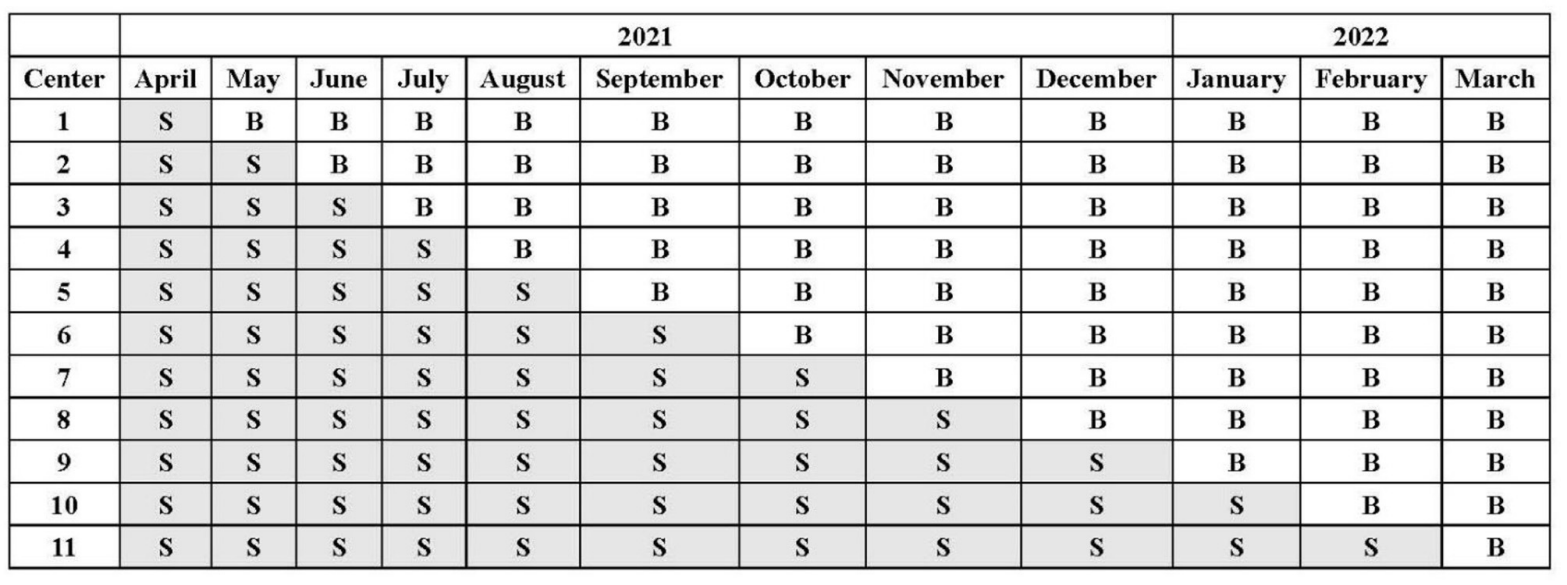
Crystalloid assignment during the trial. All sites will initiate the trial with 0.9% saline (S) as the intravenous fluid at the first month and sequentially change over to balanced crystalloids (B) without a transition period.

We used a computer-generated randomization scheme to determine the order in which each participating site crosses over from the control phase to the intervention phase. According to the stepped wedge design, all sites will initiate recruitment using NS for fluid therapy in the first month. Every month thereafter, one hospital will be randomized to use balanced crystalloids without a transition period. The assignment of patients will depend on their admission date (Figure 1).

This study is open-label. Therefore, no blinding method will be applied to the investigators. However, study participants, operators who perform the blood test, and data analysts will be masked to the allocation sequence. Study investigators will be blinded to the allocation sequence, with only the next hospital randomized for rollout being revealed a week before each transition time point.

### Sample Size, Centers, and Recruitment

According to the previous study, the standard deviation of serum chlorine in patients with severe acute pancreatitis is conservatively estimated at 6mmol/L (22). A total of 240 patients will be recruited from 11 centers within 12 months (20 patients per month). They will provide 90% power to detect the serum chlorine difference of 1.5 mmol/L or more between the balanced crystalloid and the saline group on the basis of within period intra-class correlation coefficient (ICC) of 0.05 and cluster auto-correlation (CAC) of 0.5.

The starting date of patient recruitment was Apr 1^st^, 2021, and the planned finishing date is Mar 31^st^, 2022. The follow-up will be finished after the discharge of the last recruited patient.

### Study Interventions

Study protocol determines only the choice of intravenous isotonic crystalloid: 0.9% sodium chloride or other hyperchloremic solutions (saline group) vs. the Sterofundin ISOTM (balanced crystalloid group). The compositions of each crystalloid solution are given in Additional file 1: Supplementary Table 1.

#### Group 1: Balanced Crystalloid Fluid

Patients will receive Sterofundin ISOTM (B. Braun) whenever isotonic intravenous fluid administration is ordered by the treating clinical team for seven days after enrollment or until discharge or death (whichever happens first).

#### Group 2: NS Fluid

Patients assigned to saline fluid will receive 0.9% Saline or other hyperchloremic solutions whenever isotonic intravenous fluid administration is ordered by the treating clinical team for seven days after enrollment or until discharge or death (whichever happens first).

#### Principles for Fluid Therapy

For patients who are not resuscitated before enrollment, fluid resuscitation will be initiated based on the 2013 IAP/APA guidelines (7). Briefly, 5–10 ml/kg/h of the study crystalloids should be used until resuscitation goals are reached. The resuscitation targets include one or more of the following: (1) non-invasive clinical targets of heart rate <120/min, mean arterial pressure between 65 and 85 mmHg, and urinary output >0.5ml/kg/h, (2) invasive clinical targets of stroke volume variation, and intrathoracic blood volume determination, and (3) biochemical targets of hematocrit 35–44% (7). For patients who are resuscitated before enrollment, the assessment of fluid responsiveness and the rate of crystalloid prescription will be determined by the treating physicians based on the resuscitation goals mentioned above.

### Management of Acute Pancreatitis

Patients will be managed by the local clinical team at each participating site based on the 2013 IAP/APA guidelines (7), and most clinical decisions will be left to them. All patients will receive initial standard treatment, including early enteral nutrition (start within 48 h after admission), routine medical treatment (antibiotics and sedatives as needed, organ support like mechanical ventilation, and vasopressors commenced at the discretion of the treating team. Patients with AKI (≥1.5x increase in creatinine above known baseline value) and meet predefined specific criteria described by Bellomo et al. (21) will be eligible for the introduction of RRT. Suspected infection of local pancreatic collections will be treated by the'step-up' approach, starting with percutaneous or transmural drainage followed by debridement using the preferred technique at each participating site.

### Endpoints

#### Primary Endpoint

The primary endpoint is plasma chloride concentration on the morning of day 3. The day 3 chloride will be the chloride measured in the morning bloods on the third day after enrollment, with the day of enrollment labeled day 1, the next day labeled day 2, and the following day labeled day 3. Thus, the measurement of day3 chloride will typically be 36–48 h from enrollment. The time point is chosen because high-rate intravenous fluid administration is expected during this period (initial 24–48 h), as early aggressive hydration is recommended in AP patients (23).

#### Secondary Endpoints Outcome Measures

##### Part I: Key Secondary Endpoints

MAKE 30 (Major Adverse Kidney Events by hospital discharge or day 30) and its components, including death, new receipt of RRT, or persistent renal dysfunction during the same time interval.

##### Part II: Clinical Points

1. Requirement of ICU admission during the index admission.
2. Intensive care unit free and alive days to day 30.
3. Hospital free and alive days to day 30.
4. Ventilator free and alive days to day 30.
5. Vasopressor free and alive days to day 30.
6. RRT-free and alive days to day 30.
7. New-onset organ dysfunction as judged by SOFA score [Time Frame: 30 days of enrollment].
8. Daily SOFA score from day1 to day7.
9. Daily SIRS score from day1 to day7.
10. Daily assessment of abdominal pain (VAS score) from day1 to day7.
11. Intravenous morphine equivalent dose from day1 to day7.
12. Occurrence of infected pancreatic necrosis (IPN) during the index admission.
13. Occurrence of sepsis during the index admission.
14. Occurrence of intra-abdominal bleeding during the index admission.
15. Occurrence of gastrointestinal fistula during the index admission.
16. All-cause mortality during the index admission.
17. Requirement of open surgery during the index admission.

##### Part III: Biochemical Endpoints

1. Highest creatinine in the first seven days.
2. Highest serum chloride in the first seven days of enrollment.
3. Daily serum chloride from day1 to day 7.
4. Daily serum bicarbonate from day1 to day 7.
5. Plasma NGAL concentration at day1, day2, day3, and day5.
6. Healthcare resource use (hospital expenditure) during the index admission.

### Monitored Parameters and Data Collection

**Figure 2:**
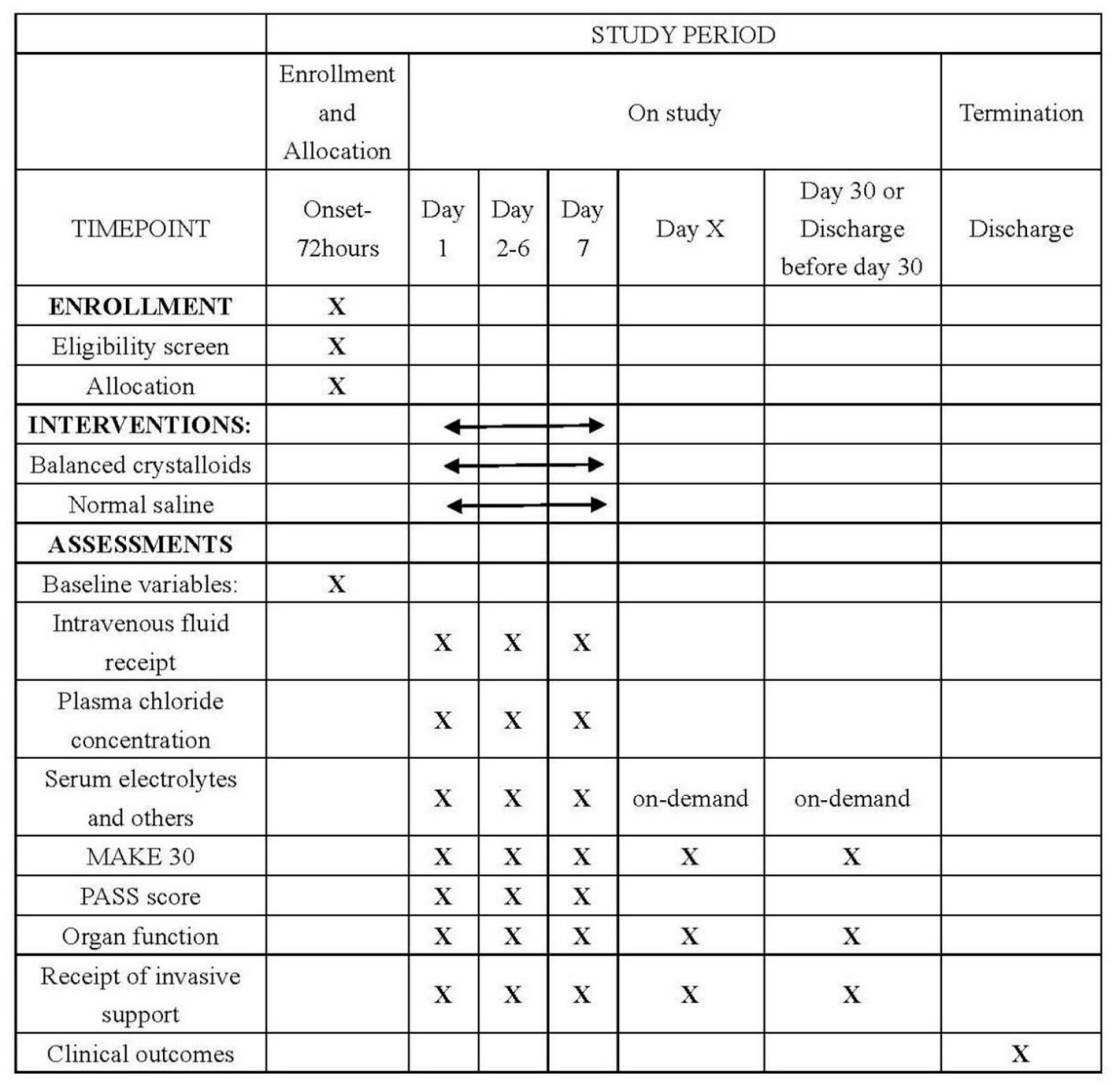
Schedule of enrollment, interventions, and assessments. MAKE denotes major adverse kidney events. PASS denotes pancreatitis activity scoring system.

A web-based electronic database (Unimed Scientific, Wuxi, China) will be used for data collection and storage. All data are inputted by the nominated investigator at each participating site. Training for data entry was arranged by the provider for all nominated investigators by the CAPCTG coordinating center before study commencement. The data required to be collected during different phases are shown in Figure 2. The CAPCTG coordinating center will be responsible for data management, safety, privacy, and quality.

### Statistical Analysis

The reporting and presentation of this trial will follow the CONSORT guidelines for stepped-wedge trials (24). Based on the principle of intention to treat (ITT), a full-analysis set (FAS) will be performed on the population with outcome reporting (day3 chloride concentration). FAS will be used for the analysis of baseline characteristics and main therapeutic interventions. The safety set (SS) will include all enrolled patients for assessing the safety profile of the intervention.

Descriptive statistics will be used to assess any marked baseline differences in demographics or outcome measures between the two groups, taking clustering into account. Comparisons of binary outcomes will be expressed as relative risk with 95% confidence intervals and comparisons of continuous outcomes as mean differences together with 95% confidence intervals. Between-group comparisons will be made using generalized linear mixed-model (GLMMs) with a hospital-level random effect to address clustering by hospital and random slopes of time to account for temporal effects, as delineated by Hussey and Hughes. Two-sided 5% significance levels will be used to identify statistically significant results. All confidence intervals reported will be 95% confidence intervals.

A series of subgroup analyses will be conducted for the evaluation of the primary endpoint: age (dichotomized at 60 years old), referral or non-referral, presence of AKI at enrollment, and presence of organ failure at enrollment.

For any possible missing data, we assume the data is missing at random while performing analyses by GLMM. Moreover, a series of sensitivity analyses after imputing the missing primary outcome using worse-case (i.e., values at baseline), Last Observation Carried Forward (LOCF), and multiply imputation will be used to validate and test the robustness of our trial result.

### Adverse Events

All the participating sites and clinical teams will be required to report all adverse events to the CAPCTG coordinating center. An independent data safety and monitoring board (DSMB) (consisting of a surgeon, an intensivist, and a statistician) will oversee all aspects of patient safety. The DSMB will review all the safety profiles regularly (every 2 months) during the study period. Adverse events (AE) will be reported in a uniform format through the electronic data capture system.

## Discussion

The best crystalloid for the early resuscitation of patients with AP remains controversial. The CLEVER-AP trial will, for the first time, provide evidence regarding the effects of normal saline and balanced crystalloid fluid on plasma chloride concentration and AKI in patients with pSAP. The findings of this trial will have direct clinical consequences as it will provide definitive evidence.

MAKE30 is identified as a key clinical endpoint, and it has been used successfully in several fluid trials (12, 25). MAKE30 is the composite of death, new RRT, or persistent renal dysfunction (PRD), which is recommended by the National Institute of Diabetes and Digestive and Kidney Diseases (NIDDK) workgroup for capturing effects of the short-term or longer-term evolution of AKI (26, 27). As such, it is a patient-centered and highly relevant endpoint for clinical research. Adopting MAKE30 as a key secondary endpoint will allow comparison with other major trials conducted with different patient populations.

The stepped-wedge cluster randomized design is adopted for the CLEVER-AP trial based on both methodology and clinical merits it could provide. From the methodology perspective, it uses the hospital as the entity of randomization because this will minimize the risk of treatment contamination and could greatly reflect the real-life practice across each hospital compared to randomization at the patient or physician level. Moreover, this design could model and assess the underlying temporal trends of the intervention with all hospitals accessing the intervention at the end of the rollout period. From the clinical perspective, since massive intravenous fluid infusion is essential and crucial in managing early acute pancreatitis, randomization at the patient level would make the intervention (fluid infusion with different types of crystalloids) vulnerable to misuse, potentially leading to contamination (12, 28). Moreover, the routine consent-and-randomization process in patient-level randomized trials takes a lot of time, during which no one knows what fluid should be used before randomization. Therefore, intravenous fluid before randomization, which could be massive, could significantly confound the trial results.

The CLEVER-AP trial is sponsored by Jinling Hospital of Nanjing University, which is the national referral center for AP, admitting more than 800 cases of AP annually. The trial will be coordinated by the CAPCTG coordinating center (capctg.medbit.cn), which is capable of and experienced in conducting nationwide trials (29, 30).

In conclusion, the CLEVER-AP trial aims to evaluate the effects of balanced crystalloids vs. NS on plasma chloride concentration, major adverse kidney events, and clinical outcomes in predicted severe acute pancreatitis patients.

## Strengths and Limitations

### Strengths

1. This is a stepped-wedge cluster-randomized, multicenter, controlled trial providing high-level evidence about the impact of NS and a balanced crystalloid on plasma chloride concentration and renal function/AKI/renal failure, a significant consequence of severe AP.
2. The data will be reviewed by an independent data safety monitoring board (DSMB) to ensure the participants' safety.

### Limitations

1. Mild acute pancreatitis patients who may also benefit from the use of balanced crystalloids will not be included;
2. The study sample size is relatively small and not sufficiently powered to detect a difference in patient-centered outcomes like MAKE 30.

### Trial Status

The CLEVER-AP trial will be conducted between Apr 1^st^, 2021, to Mar 31^st^, 2022.

## Ethics Statement

The studies involving human participants were reviewed and approved by the Chinese Clinical Trials Registry (ChiCTR2100044432). Written informed consent for participation was not required for this study in accordance with the national legislation and the institutional requirements.

## Author Contributions

All authors were involved in the study design, and read and approved the final manuscript. During the study, BY, MH, and TC were responsible for randomizing the patients and ensuring the blinding. BY, MH, YC, BW, MC, ST, XC, MY, GZ, QL, XP, LZ, and HX were responsible for carrying out recruitment, managing the treatment of the patients and collecting data. BW, MC, ST, XC, MY, GZ, QL, XP, LZ, and HX are members of the Trial Steering Committee. LK, ZT, TC, GD, JW, RB, and WL drafted the manuscript. All authors contributed to the article and approved the submitted version.

## Funding

The study was funded by the Key Research and Development Program Foundation of Jiangsu Province of China (No. BE2016749), Young Scientists Fund of the National Science Foundation of China (81900593) and partly by the Jiangsu Nhwa Pharmaceutical Co., Ltd. The funders had no role in the study's design, data collection, and interpretation, preparation, and publication of the manuscript.

## Conflict of Interest

The authors declare that the research was conducted in the absence of any commercial or financial relationships that could be construed as a potential conflict of interest.

## Publisher's Note

All claims expressed in this article are solely those of the authors and do not necessarily represent those of their affiliated organizations, or those of the publisher, the editors and the reviewers. Any product that may be evaluated in this article, or claim that may be made by its manufacturer, is not guaranteed or endorsed by the publisher.
