# Supplementary material for: The Impact of Normal Saline or Balanced Crystalloid on Plasma Chloride Concentration and Acute Kidney Injury in Patients With Predicted Severe Acute Pancreatitis: Protocol of a Phase II, Multicenter, Stepped-Wedge, Cluster-Randomized, Controlled Trial
Source: Front Med (Lausanne). 2021 Oct 4;8:731955. doi: 10.3389/fmed.2021.731955 (PMC8521113; doi:10.3389/fmed.2021.731955)
Supplement: Supplementary file 1 [file Table_1.docx]

| **Compositions** | **Sterofundin ISOTM** | **Normal saline** |
| --- | --- | --- |
| **Na+** | 145.0mmol/L | 154.0mmol/L |
| **K+** | 4.0mmol/L | - |
| **Ca2+** | 2.5mmol/L | - |
| **Mg2+** | 1.0 mmol/L | - |
| **Cl-** | 127.0 mmol/L | 154.0 mmol/L |
| **Acetate** | 24.0 mmol/L | - |
| **Malate** | 5.0 mmol/L | - |

**Table S1: The compositions of each crystalloid solution.**
